# Survey of knowledge and attitude regarding induced abortion among nurses in a tertiary hospital in Thailand after amendment of the abortion act: a cross-sectional study

**DOI:** 10.1186/s12905-022-02064-7

**Published:** 2022-11-18

**Authors:** Natchanika Sinthuchai, Penkae Rothmanee, Vorachart Meevasana, Ratthapong Rongkapich, Rada Poolkumlung, Saowanee Saro, Somsook Santibenchakul, Unnop Jaisamrarn

**Affiliations:** 1 Obstetrics and Gynecology Unit, Nakhon Pathom Hospital, 196 Tesa Road, Phra Pathom Chedi, Mueang Nakhon Pathom, Nakhon Pathom, 73000 Thailand; 2 Obstetrics and Gynecology Unit, Naradhiwasrajanagarindra Hospital, 180 Ra-ngae Mankha, Bang Nak, Mueang, Narathiwat, 96000 Thailand; 3 Obstetrics and Gynecology Unit, Pimai Hospital, 138 Pimai Road, Amphoe Phimai, Nakonratchasrima, 30110 Thailand; 4 Faculty of Medicine, Chulalongkorn University, 1873 Rama IV Rd, Pathum Wan, Pathum Wan District, Bangkok, 10330 Thailand; 5 Obstetrics and Gynecology Unit, Sungaikolok Hospital, 1 Saithong 5 Road, Sungaikolok, Narathiwat, 96120 Thailand; 6 Department of Obstetrics and Gynecology, King Chulalongkorn Memorial Hospital, 1873 Rama IV Rd, Pathum Wan, Pathum Wan District, Bangkok, 10330 Thailand; 7 Department of Obstetrics and Gynecology, Faculty of Medicine, Chulalongkorn University, 1873 Rama IV Rd, Pathum Wan, Pathum Wan District, Bangkok, 10330 Thailand

**Keywords:** Knowledge, Attitude, Nurses, Thai abortion law, Muslim, Induced abortion, Unwanted pregnancy

## Abstract

**Background:**

The abortion act in Thailand is approximately 60 years old. However, because of increasing problems due to unsafe abortions, the act was recently amended to accord a legal status for abortions. In the southernmost provinces of Thailand, most people follow the Islamic faith, according to which induced abortion is a sin for both the providers and the pregnant women. This may affect the attitude of the medical staff, such as registered nurses, who play an important role in abortion services. Our study aims to evaluate the knowledge of the amended abortion act, attitude toward abortions and the intentions behind them, and willingness to perform abortions among registered nurses.

**Methods:**

A cross-sectional study was conducted from January 2022 to February 2022 wherein a self-administrated questionnaire was electronically distributed to 450 registered nurses practicing at a tertiary hospital in the southernmost province of Thailand. Linear regression analysis and Fisher’s exact test were conducted to evaluate the association between basic characteristics, knowledge scores, and attitudes toward induced abortion.

**Results:**

A total of 375 nurses (83.3%) completed the survey. Most participants were Muslim (58.9%), and 18.7% of them correctly answered > 80% of the knowledge questions. Among all the participants, 41.4% had a favorable attitude toward induced abortion, of which 21.3% were willing to provide safe abortion services. Knowledge scores were independently associated with practicing in obstetrics-gynecology departments and a lower age. Participants practicing Buddhism and having good knowledge scores tended to have favorable attitudes toward abortion.

**Conclusions:**

Nurses in the southernmost province of Thailand lack knowledge regarding the amended abortion act and do not have a favorable moral attitude toward abortion. Favorable attitudes toward abortions, support toward intentions behind abortions, and a willingness to provide abortion services were all lesser among the Muslim participants than among the Buddhist participants. Compared with participants who scored lower, those with higher knowledge scores had a better moral attitude toward abortion and, in turn, demonstrated a greater intention to provide abortion services. Encouraging nurses to gain better knowledge may improve their attitude toward abortion, which may positively influence future medical practices.

**Supplementary Information:**

The online version contains supplementary material available at 10.1186/s12905-022-02064-7.

## Background

Unplanned pregnancy is one of the most serious public health issues. It not only affects the physical and mental health of the mother and child [1, 2] but also results in economic and social problems [3, 4]. According to Thailand’s Foundation for Women’s Health and Reproductive Rights, the abortion prevalence in Thailand is 300,000 per year [5]. In 2020, survey data obtained by the Bureau of Reproductive Health in Thailand revealed that induced abortions accounted for 53.8% of all abortions [6], most of which were because of unwanted pregnancies, and 3.2% among them resulted in serious complications due to unsafe abortion procedures [6].

Unsafe abortion can lead to complications and death [7–9]. The overall maternal mortality rate was 211 per 100,000 livebirths in 2017, 8% of which resulted from abortion [10]. The mortality rate from unsafe abortions in developing countries were 220 deaths per 100,000 unsafe abortions [11], while the mortality rate from legal abortions was around 0.6 deaths per 100,000 legal abortions [12]. Evidence from a previous study shows that abortions in countries with more legal restrictions were more likely to be unsafe than those in countries with fewer restrictions [13, 14].

In Thailand, prior to the amendment of abortion laws, safe abortion services were very limited, as abortion was illegal except when performed by a physician in circumstances in which the pregnancy was harmful to the woman’s health or was the result of a sexual crime. Based on worldwide trends of the liberalization of abortion laws, evidence regarding the negative impact of unsafe abortions [13, 14], and several problems from the interpretation and implementation of the previous Thai law, the Amended Act of the Penal Code (no. 28) was announced on February 5, 2021 [15]. In this amended law, punishment was withheld under the following conditions: 1) the continuation of the pregnancy threatened the woman’s physical or mental health, 2) fetal anomalies incompatible with life were present, 3) the pregnancy resulted from a sexual crime, 4) the gestational age was < 12 weeks, and 5) the gestational age was 12–20 weeks in pregnant woman with a strong desire to undergo abortion following a thorough medical consultation [15]. The combipack of mifepristone and misoprostol, available in Thailand since 2014, combined with the amended abortion laws, has changed the abortion practice in Thailand.

Medical abortion has been increasingly used and accepted by healthcare providers and patients. Nowadays, primary care settings, including both public and private sectors, provide safe abortion services. Only complicated cases, such as women with medical conditions or gestational ages > 12 weeks, are referred to secondary and tertiary settings. However, the referral system in Thailand has not been well established, and these vulnerable groups have faced improper care. For example, the waiting time may be too long, extending until the gestational limit for abortion has passed. Abortion is an extremely sensitive issue, and the decision to provide safe abortion services may be influenced by not only legal factors but also by religious ones [16]. In 2011, Harris et al. [17] found that obstetricians in the United States who were Protestant Christians, Roman Catholics, and Muslims objected to abortion. However, two-thirds of participants were willing to perform abortions. Other studies also showed that Muslim healthcare providers have a negative attitude toward abortion [18, 19]. A study by Rehan et al. [18] revealed that a majority of healthcare providers in Pakistan (67.3%) have a negative attitude toward abortion, with 37.7% of them having the opinion that abortion laws should be more stringent.

According to Islamic criminal law, the main elements of abortion offenses are the act, the pregnant woman, and the intention [20]. The gestational age at the time of abortion is an essential factor in determining penalties in Islamic criminal law [20]. To date, there is no consensus among Islamic scholars regarding the gestational age beyond which abortion would be considered an offense. Some indicate that inducing an abortion after 120 days of gestation is a punishable offense [20]. Under Islamic criminal law, the person(s) involved in performing the abortion, including the pregnant woman, the husband, the doctor, and the nurse, may be punished [20].

Registered nurses provide services related to family planning and contraception, including providing abortion-related counseling, assisting physicians to perform abortions, and delivering patient care before and after the abortive procedure, in most public healthcare settings including at primary and secondary levels [21, 22]. In Thailand, only physicians can legally perform abortions. However, nurses play a significant role in providing abortion services, such as counseling and assisting during surgery. Therefore, their attitude toward abortion and willingness to assist with the procedure can affect patient access to safe abortion services [23]. In 2017, Ismail et al. [24] pointed out that nurses in Malaysia are key persons who help patients make decisions and also participate in abortion procedures. Therefore, nurses require additional education and knowledge of the laws regarding abortion to help them make moral decisions related to abortions.

The impact of the amended abortion laws, which seem to contradict Islamic religious principles, on reproductive health services in Thailand has not been evaluated. Therefore, our primary objective was to study the impact of religion on abortion knowledge regarding the amended abortion act. Our secondary objectives were to study the impact of religion on abortion attitudes and willingness to provide abortion services. We assessed the knowledge regarding the amended abortion laws as well as an attitude toward abortions and the intentions behind them among registered nurses in the southernmost province of Thailand, where the largest numbers of the Muslim population in Thailand are located. We aim to use this knowledge as a basis for the future development of Thailand’s reproductive health planning schemes. This data may be helpful for nearby South East Asian countries with similar situations.

## Methods

### Sample selection and size

A cross-sectional survey was conducted to evaluate the knowledge regarding the amended abortion act as well as the attitude toward induced abortions and intentions behind them among nurses at one of the tertiary hospitals (400 beds hospital and the largest referral center among the three southernmost provinces of Thailand) in the southernmost province of Thailand, which is considered to have one of the largest Muslim communities in the country. From January to February 2022, self-administered, web-based questionnaires were distributed to 450 registered nurses (all registered nurse in the hospital was recruited) at the study site. We used Research Electronic Data Capture tools hosted at Chulalongkorn University to collect the data [25, 26].

### Questionnaires

Each questionnaire comprised the following five categories of questions: baseline characteristics, including, demographic data, such as sex, age, marital status, religion, and years of nursing experience; knowledge of the amended abortion act (10 true/false questions), which was a modified questionnaire published by Bunnag et al. in 2016 [27], that assessed knowledge of the former Thai abortion law by incorporating questions related to the amended abortion acts, from sections 301 and 305, published in the Government Gazette on February 6, 2021 [15]; moral attitude toward abortion (seven questions); attitude toward intentions behind abortion (26 questions); and willingness to provide abortion services, including the approach adopted in managing patients when abortion services were denied. The questionnaire regarding knowledge of the amended abortion act was tested for internal consistency and reliability using Cronbach’s alpha coefficient (0.7) and test-retest reliability (0.9). Furthermore, considering the gestational age as a significant factor that determines whether abortion is a punishable offense, the questions were categorized into the following groups: abortion before 12 weeks of pregnancy and abortion after 12 weeks of pregnancy. The questions regarding moral attitude and intentions were modified from a previous study [27] and derived, with permission, from a study published by Baba et al. [28], translated to Thai, and approved by two family planning experts (SS and UJ); the Cronbach’s alpha coefficient, and the test-retest reliability values for both the moral attitude and intentions parts were 0.8 and 0.9, respectively.

### Survey administration

The official documents, including a description of the study and an invitation for participation, were distributed to the hospital director. Subsequently, the project was introduced and described to each participant using the LINE communication application [29]. After the nurses provided consent to participate in the survey, the questionnaires were anonymously administered via an online link, which was accessible either through mobile devices or computers. The participants were able to complete the questionnaire in 15–20 minutes, after which each of them received a compensation of 100 THB (approximately 3.5 USD) for their time.

### Outcome variables

In the knowledge score section, participants with scores > 80% were considered to have good knowledge. In the moral attitudes section, the statements pertaining to pro-choice and conditional agreement were assigned a score of 1 for “strongly disagree” and 5 for “strongly agree,” while it was vice versa for the pro-life statements. A mean attitude score was then calculated and used as a cut-off point for the moral attitude favoring abortion.

### Statistical analysis

All statistical analyses were performed using IBM SPSS Statistics for Windows, version 22.0 (IBM Corp., Armonk, NY, USA). Continuous variables are represented as mean (standard deviation [SD]) and categorical variables as numbers and percentages. Linear regression with 95% confidence interval was used to evaluate the association between demographic variables and knowledge score. We adjusted for potential confounders by variables with *p* <  0.05. All *p*-values < 0.05 were considered to indicate a statistically significant difference. The associations of demographic variables with the attitude toward abortion and intention behind abortion were assessed using the Fisher’s exact test.

## Results

### Baseline characteristics

Among 450 registered nurses, 375 completed the questionnaire, yielding a response rate of 83.3%. Approximately 60% of the participants were Muslim, and 40% were Buddhist. The basic characteristics of participants are shown in Table 1. Compared to Buddhist participants, Muslim participants were younger (*p* <  0.05), married (*p* <  0.05), and had less experience working as a part of a team in caring for unintended pregnancies (*p* <  0.05). The mean (SD) number of years spent practicing as a registered nurse among the Muslim participants was 12.3 (6.8) years, while it was 19.4 (8.1) years among the Buddhist participants (*p* < 0.05). Furthermore, 33% of Muslim participants and 47% of Buddhist participants had experience providing safe abortion services.

**Table 1:** Baseline characteristics of nurses by religion (*N* = 375)

Variable	Muslims	Buddhists	Total	***P***-value
**Age –** Mean (SD)	36.6 (6.9)	42.8 (7.0)	39.2 (7.6)	< 0.01
**Age group -** No. (%)				< 0.001
20–29 years	24 (10.9)	2 (1.3)	26 (6.9)	
30–39 years	133 (60.2)	44 (28.6)	177 (47.2)	
40–49 years	53 (24.0)	83 (53.9)	136 (36.3)	
more than 50 years	11 (5.0)	25 (16.2)	36 (9.6)	
**Sex -** No. (%)				0.647
Male or Transmen	8 (3.6)	7 (4.5)	15 (4.0)	
Female or Transwomen	210 (94.9)	146 (94.8)	356 (94.9)	
Others	1 ((0.5)	1 (0.6)	2 (0.5)	
Prefer not to say	2 (0.5)	0 (0.0)	2 (0.5)	
**Marital status -** No. (%)				0.001
Single	60 (27.1)	37 (24.0)	97 (25.9)	
Married or cohabitation	149 (67.4)	91 (59.1)	240 (64.0)	
Divorced/Widow	12 (5.4)	26 (16.9)	38 (10.1)	
**Having Child/Children -** No. (%)				0.508
No	80 (36.2)	50 (32.5)	130 (34.7)	
Yes	141 (63.8)	104 (67.5)	245 (65.3)	
**Regions of living during childhood -** No. (%)				0.114
Central	0 (0.0)	0 (0.0)	0 (0.0)	
Northern	0 (0.0)	2 (1.3)	2 (0.5)	
Southern	221 (100.0)	151 (98.1)	372 (99.2)	
North-Easten	0 (0.0)	1 (0.6)	1 (0.3)	
Eastern	0 (0.0)	0 (0.0)	0 (0.0)	
Western	0 (0.0)	0 (0.0)	0 (0.0)	
**Department of practicing** – No. (%)				0.350
OB-GYN	67 (30.3)	38 (24.7)	105 (28.0)	
Others	148 (67.0)	109 (70.8)	257 (68.5)	
Not defined	6 (2.7)	7 (4.5)	13 (3.5)	
**Team experience caring for unintended pregnancies or abortions** – No. (%)				0.005
Yes	73(33.0)	73(47.4)	146(38.9)	
No	148(67.0)	81(52.6)	229(61.1)	
**Number of years spent practicing as a registered nurse –** Mean (SD)	12.3 (6.8)	19.4 (8.1)	15.2 (8.1)	< 0.05

^a^Others included non-binary, gender fluidity and agender

Considering the religion of the participants, a majority of the Muslim participants belonged to the Sunni branch of Islam and practiced *Salat* five times a day (94%), while only approximately 14% of the Buddhist participants prayed more than five times a week. Details of this religiosity are shown in Table 2.

**Table 2:** Religion and religiosity of participants (*N* = 375)

Variable	Frequency – No. (%)
**Islam**	**221 (58.9)**
**Sect**	
Sunni	207 (93.7)
Shia	4 (1.8)
Others	10 (2.7)
***Salat***	
5 times/day	208 (94.1)
1–4 times/day	10 (4.5)
Sometimes	2 (0.9)
Never or seldom	1 (0.5)
***Saum***	
Whole month	180 (81.4)
16–27 days	37 (16.7)
8–15 days	3 (1.4)
< 7 days	0
Not observing *Saum*	1 (0.5)
***Zakat*** **charity**	
Yes	220 (99.5)
No	1 (0.5)
**Buddhism** – No.(%)	**154 (41.1)**
**Pray**	
5–7 times/week	21 (13.6)
1–4 times/week	44 (28.6)
Buddhist-day	10 (6.5)
Never or seldom	79 (51.3)
***Takbat*** (To offer food to the monks)	
5–7 times/week	2 (1.3)
1–4 times/week	30 (19.5)
Buddhist-day	12 (7.8)
Never or seldom	110 (71.4)
**Practice** ***Pancha Sila***	
5–7 times/week	37 (24.0)
1–4 times/week	53 (34.4)
Buddhist-day	12 (7.8)
Never or seldom	52 (13.9)
**Meditation**	
5–7 times/week	7 (4.5)
1–4 times/week	24 (15.6)
Buddhist-day	7 (4.5)
Never or seldom	116 (75.3)

### Knowledge

**Fig. 1:**
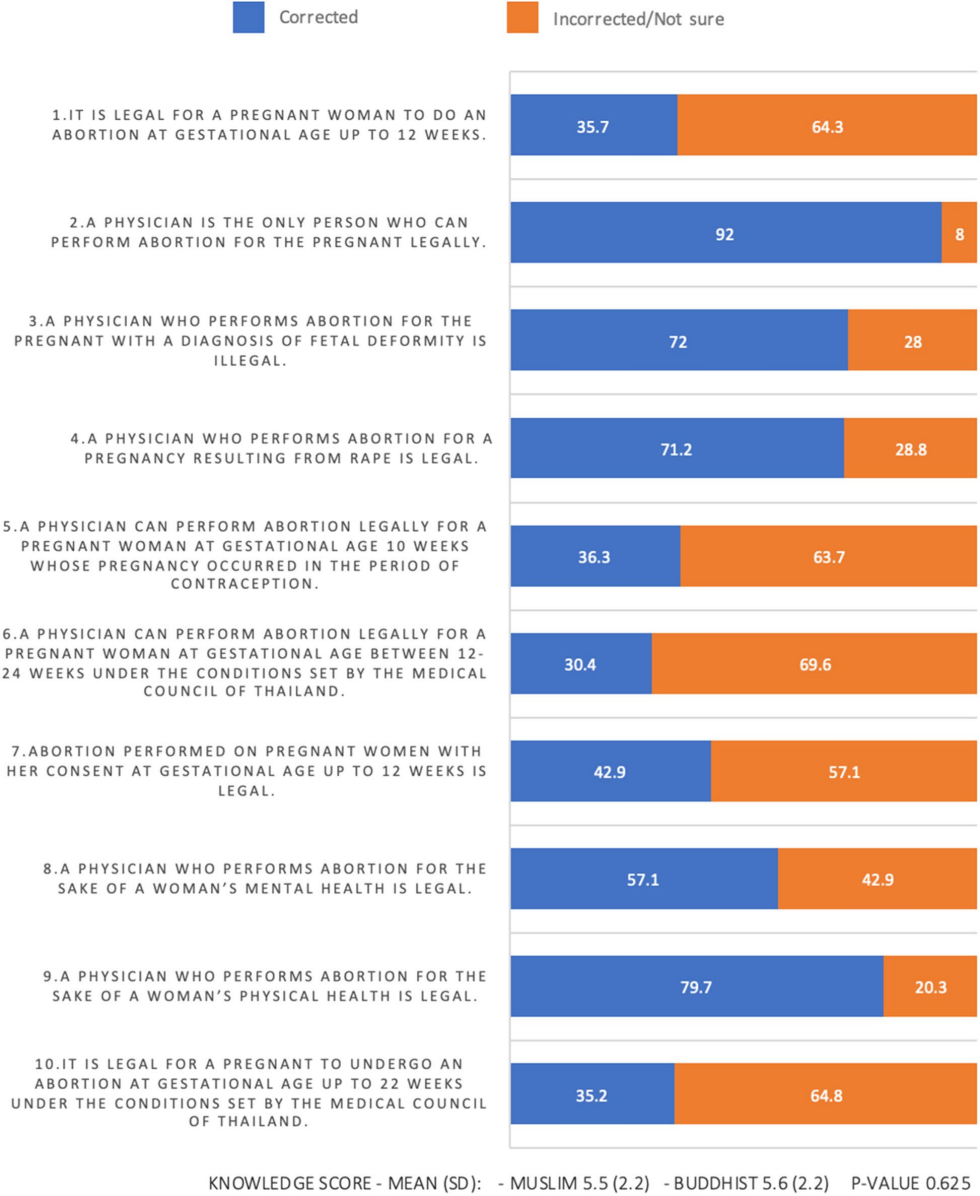
Percentage of Corrected Responses to Each Question (*N* = 375)

The mean (SD) knowledge scores of the Muslim and Buddhist participants were 5.5 (2.2) and 5.6 (2.2) points, respectively. Only 19.4% of the Muslim participants and 17.5% of Buddhist participants correctly answered at least 80% of the knowledge questions. Considering questions on the amended abortion act (question numbers 5, 6, 7, and 10), 30–40% of the participants recognized this amendment (Fig. 1). Regarding the knowledge that the amended abortion act was not very different from the previous version (question numbers 3, 4, 8, and 9), 60–70% of the participants correctly answered these questions. Higher knowledge scores were associated with a lower age, fewer years practicing as a registered nurse, and currently working in the OB-GYN department, with *p*-values of < 0.01, 0.02, and < 0.01, respectively. In multivariable analysis, we found that the participants’ knowledge scores were independently associated with age, number of years practicing as a registered nurse, and currently working in the OB-GYN department (Table 3).

**Table 3:** Linear regression models of participants’ knowledge of the amended Thai abortion act

Demographics	Knowledge score ranging from 0 to 10 points			Model I^**c**^		Model II^**c**^	
	Mean	Mean difference (95% CI^**a**^)	***p***-value	Adjusted mean difference (95% CI^**a**^)	***p***-value	Adjusted mean difference (95% CI^**a**^)	***p***-value
**Age group**			< 0.01*		< 0.01*		
20–29 years	7.19	Reference					
30–39 years		−1.70 (−2.58,-0.82)	< 0.01	−1.62 (−2.47,-0.77)	< 0.01		
40–49 years		−1.88 (−2.77,-0.98)	< 0.01	−1.70 (−2.57,-0.83)	< 0.01		
more than 50 years		−1.94 (−3.02,-0.87)	< 0.01	−1.72 (− 2.77,-0.68)	< 0.01		
**Gender**			0.75*				
Female/ Trans women	5.53	Reference					
Others^**b**^		−0.17 (− 1.17,0.84)	0.75				
**Marital status**			0.14*				
Single	5.81	Reference					
Married or cohabitation		−0.46 (−0.97,0.06)	0.08				
Divorced/Widow		0.03 (−0.79,0.84)	0.95				
**Having Child/Children (%)**			0.08*				
No	5.79	Reference					
Yes		−0.41 (− 0.87,0.05)	0.08				
**Religion**			0.63*				
Buddhist	5.59	Reference					
Muslim		−0.11 (−0.56,0.34)	0.63				
**No. of years in practicing as a nurse – years**	6.04	−0.03 (− 0.06,-0.01)	0.02*			−0.03 (− 0.06,0.00)	0.03
**Department**			< 0.01*				* **< 0.01**
OB-GYN	5.21	1.24 (0.76,1.17)	< 0.01	1.17 (0.70,1.64)	< 0.01	1.20 (0.72,1.67)	< 0.01
Others		Reference					
Not defined		−0.90 (−2.07,-0.27)	0.13	−0.93 (−2.08,0.23)	0.12	− 0.92 (−2.08,0.24)	0.12
**Experience in taking care of unintended pregnancy or abortion**s			0.10*				
Yes		0.37 (−0.08,0.82)					
No	5.38	Reference	< 0.10				

^a^*CI* confidence interval

^b^ Others included sex other than female/transgender women

^c^ Model I: age group and department, Model II: number of years practicing as nurses and department

* *P* model

### Moral attitude towards abortion

Questions regarding the moral attitude of the participants toward abortion were divided into pro-life, pro-choice, and conditional agreement sections. Regarding moral attitude statements in the pro-choice and conditional agreement section, except for the statement of “abortion is a woman’s right” and “abortion after gestational age 12 week is acceptable in some situation,” very few Muslim and Buddhist participants stated that they agree/strongly agree with the statements. However, most Muslim and Buddhist participants stated that they agree/strongly agree with the moral attitude statements in the pro-life section. (See Supplemental Table 1, Additional file 1) The attitudes toward intentions behind abortion were divided into a gestational age < 12 weeks and > 12 weeks groups. The results were comparable between both groups. (See Supplemental Table 2, Additional file 1) Both the Muslim and Buddhist participants tended to agree to perform an abortion under the following conditions (mentioned in decreasing order of number of participants): the fetus has a serious defect, the pregnant woman has a serious physical disease, and the pregnant woman has a serious mental disease. Very few participants were likely to provide safe abortion services to women with socioeconomic problems.

### Willingness to provide abortion services

Only 21.3% of the participants were willing to provide safe abortion services, with the Buddhist participants (26%) showing more willingness than the Muslim participants (18.1%). (See Supplemental Table 3, Additional file 1) The participants willing to provide safe abortion services agreed to provide either a medical or surgical abortion. Among the participants unwilling to provide safe abortion services, the majority of the Muslim participants (48.1%) stated that they would advise women to continue the pregnancy and look into adoption processes. In contrast, most of the Buddhist participants (58.8%) in this category preferred referring the women for safe abortion services. Less than half of the participants stated that they knew where to recommend women with unintended pregnancies for safe abortion services.

### Factors associated with moral attitude and intended safe abortion practice

The Buddhist participants tended to have a more favorable moral attitude toward abortion than the Muslim participants. (Table 4) Participants with knowledge scores ≥80% tended to have a more favorable moral attitude toward abortion than those whose scores were < 80%. Data showed the association between religion, knowledge, and attitude scores related to safe abortion practices. (Table 5) Compared with the Muslim participants, the Buddhist participants tended to agree more to providing safe abortion services. Furthermore, participants who had a favorable moral attitude toward abortion (moral attitude score > mean attitude score) tended to agree more to providing safe abortion services than those with a less favorable moral attitude toward abortion.

**Table 4:** Religion and knowledge associated with moral attitude in percentage (*N* = 375)

Conditions	Religion		Knowledge	
	Muslim (***n*** = 221)	Buddhist (***n*** = 154)	More than 80% (***n*** = 70)	Less than 80% (***n*** = 305)
**Prochoice** (strongly agree and agree)				
Abortion can be a good thing in any circumstances.	5.0	9.1	10.0	5.9
Abortion is a woman’s right.	27.6*	46.8*	55.8*	30.0*
Abortion after gestational age 12 week is acceptable in every situation.	12.2	16.2	17.1	13.1
**Conditional agreement** (strongly agree and agree)				
Abortion after gestational age 12 week is acceptable in some situation.	57.5	66.2	81.4*	56.4*
**Prolife** (strongly disagree and disagree)				
Abortion is the same as murder.	15.8*	26.6*	27.1	18.7
Abortion is wrong.	17.2*	27.9*	27.1	20.3
Abortion is sinful.	10.4*	18.2*	24.3*	11.1*

*Fisher’s exact test *p* < 0.05

**Table 5:** Religion, knowledge and moral attitude associated with intended safe abortion practice (*N* = 375)

	Religion		Knowledge		Attitude	
	Muslim (***n*** = 221)	Buddhist (***n*** = 154)	More than 80% (***n*** = 70)	Less than 80% (***n*** = 305)	More than mean attitude^a^ (***n*** = 190)	Less than mean attitude^a^ (***n*** = 185)
**Circumstances (Gestational age under 12 weeks)**						
1. The pregnant woman has a serious physical disease(s).	76.9	79.9	95.7*	74.1*	86.3*	69.7*
2. The pregnant woman has HIV/ AIDS.	30.3*	45.5*	48.6*	33.8*	43.7*	29.2*
3. The pregnant woman has a serious mental disease(s).	72.4	76.2	92.9*	69.5*	82.1*	65.4*
4. The fetus has a serious defect that makes it nonviable.	80.5	87.0	94.3*	80.7*	88.9*	77.3*
5. The fetus has a serious defect but will be viable and being handicapped.	63.8*	83.8*	84.3*	62.9*	78.9*	64.9*
6. The woman has become pregnant as a result of being raped.	56.1*	72.7*	88.6*	57.0*	71.1*	54.6*
7. The woman has become pregnant as a result of incestuous pregnancy (incest - sexual intercourse between two members of the same family).	32.6*	46.8*	48.6	36.1	44.7*	31.9*
8. The pregnant woman is under age 20.	6.8	19.5	15.7	11.1	19.5*	4.3*
9. The pregnant woman is under age 15.	17.2*	27.9*	41.4*	17.0*	27.9*	15.1*
10. The man involved in the pregnancy will not support the woman in having a baby.	6.3*	15.6*	17.1*	8.5*	15.8*	4.3*
11. The man involved in the pregnancy will not marry the woman.	5.4*	12.3*	11.4	7.5	12.1*	4.3*
12. The woman/couple feels they already have enough children.	5.0*	14.9*	11.4	8.5	16.3*	1.6*
13. The woman has become pregnant as a result of contraceptive failure.	7.2*	21.4*	20.0	11.5	20.5*	5.4*
**Circumstances (Gestational age greater than 12 weeks)**						
1. The pregnant woman has a serious physical disease(s).	70.1	77.9	92.9*	68.9*	83.2*	63.2*
2. The pregnant woman has HIV/ AIDS.	38.0*	50.6*	54.3*	40. 7*	53.2*	33.0*
3. The pregnant woman has a serious mental disease(s).	63.8*	76.6*	87.1*	64.9*	78.9*	58.9*
4. The fetus has a serious defect that makes it nonviable.	75.1*	88.3*	92.9*	77.7*	87.4*	73.5*
5. The fetus has a serious defect but will be viable and being handicapped.	60.6*	83.1*	80.0*	67.5*	77.4*	62.2*
6. The woman has become pregnant as a result of being raped.	46.2*	67.5*	78.6*	49.5*	64.2*	45.4*
7. The woman has become pregnant as a result of incestuous pregnancy (incest - sexual intercourse between two members of the same family).	29.4	38.3	47.1*	29.8*	40.0*	25.9*
8. The pregnant woman is under age 20.	5.4*	16.9*	15.7	8.9	15.8*	4.3*
9. The pregnant woman is under age 15.	14.5	22.7	27.1*	15.7*	24.7*	10.8*
10. The man involved in the pregnancy will not support the woman in having a baby.	5.0*	16.9*	12.9	9.2	16.8*	2.7*
11. The man involved in the pregnancy will not marry the woman.	5.4*	16.2*	10.0	9.8	16.3*	3.2*
12. The woman/couple feels they already have enough children.	5.4*	16.2*	8.6	10.2	16.8*	2.7*
13. The woman has become pregnant as a result of contraceptive failure.	5.9*	23.4*	15.7	12.5	20.5*	5.4*

*Fisher’s exact test *p* < 0.05

^a^ mean attitude score was 16.7

## Discussion

To the best of our knowledge, this is the first study conducted on registered nurses in the southernmost province of Thailand to assess their knowledge and moral attitude toward abortion as well as willingness to provide safe abortion services after the amendment of the abortion laws. A majority of the participants lacked knowledge regarding the recently amended abortion act, and only 30% of the participants were aware of the change in the gestational age limit for legal abortions, which may be attributed to the recent announcement of the amended abortion act (approximately 10 months before conducting this study). However, this could also indicate the lack of effective communication between policymakers and healthcare personnel.

The mean knowledge scores were similar among the Buddhist and Muslim participants. Nevertheless, considering that the previous abortion act was implemented more than 60 years ago and modified by the Medical Counsel of Thailand over 16 years ago [30], a higher percentage of correct answers than that obtained in this study was expected. Furthermore, participants with higher knowledge scores were more likely to exhibit a favorable attitude toward abortion than the ones who scored less. The low knowledge score pertaining to both the previous and amended abortion act indicates that there should be an emphasis on the continuation of education by providing sessions or short training courses, as demonstrated by Sanitya et al. [31] who found that the attitude of healthcare providers toward abortion significantly improved after receiving training classes on safe abortion practices. This could assure healthcare providers that they are acting in accordance with the laws.

The results of this study are comparable to those of previous studies conducted to assess the knowledge of healthcare providers regarding abortion laws. Sanitya et al. [31] showed that 52.5% of the study population, including physicians, pharmacists, and other healthcare providers, exhibited knowledge about abortion regulations in Thailand. Afhami et al. [19] showed that in Iran, a country that legally restricts abortions, only 0.5% of midwives had a knowledge score > 80%, despite exhibiting a favorable attitude toward abortion. Our study findings demonstrated a significant negative correlation between the age of the nurses and knowledge scores (*p* < 0.01), which may be because younger nurses perform more general tasks, which may include abortion services. There was a significant association between practicing in the OB-GYN department and higher knowledge scores (*p* < 0.01), which could be attributed to a familiarity with providing safe abortion services.

Regarding the attitude toward abortion, the mean attitude scores showed that most participants did not have a favorable attitude toward abortion. They tended to disagree with pro-choice and conditional agreement statements, while they agreed on pro-life statements. These results were inconsistent with those of a study conducted in Turkey, which showed that nursing students showed moderate support toward abortion [32]. A study conducted in Ireland by Fitzgerald et al. [33] showed that 72.8% of the respondents moderately/strongly supported the pro-choice statements. The results of our study were consistent with a study conducted in Pakistan, an Asian country, by Rehan et al. [18], which showed that 67.3% of healthcare providers, including house officers, nurses, and physicians, had unfavorable attitudes toward induced abortion. These results might be because of the differences in culture and abortion regulations between Europe and Asia [34]. We also found that Buddhist participants had more favorable attitudes toward abortion, which can be explained in light of Islamic criminal law [20].

Considering the willingness of the participants to provide safe abortion services, the results were consistent between the gestational age < and > 12 weeks groups. The majority of participants agreed that a serious fetal defect warranted abortion, while the least priority was given to socioeconomic problems as a reason for providing abortion services. Our results were consistent with those of a study by Saengruang et al. [35], which showed that most new medical graduates in Thailand agreed to perform abortions based on health conditions of the mother and fetus and not socioeconomic problems. This may be because making decisions to provide abortion services in cases where the fetus is nonviable or the mother’s health/life is under threat is uncomplicated. On the contrary, socioeconomic problems do not have any apparent effect on the maternal or fetal life during pregnancy. Nevertheless, an unintended pregnancy has significant impacts, resulting in an increased risk of maternal depression and parenting stress [2], which is a major reason behind women opting for unsafe abortion procedures [6]. Participants with good knowledge scores and a favorable attitude toward abortion expressed their intention to provide safe abortion services. This was consistent with a study conducted in Poland by Michalik et al. [36], which showed that midwifery graduates in the final stages of a university education accepted and asserted their willingness to perform abortions. Thus, receiving training for abortion procedures may improve the attitude toward it.

We also found that only a small number (21.3%) of participants were willing to perform abortions. In contrast, Enyew et al. [37] showed that 68.4% of participants of a study conducted in Ethiopia expressed a willingness to perform abortions, which was affected by religious practices. However, the participants were nursing, midwifery, and medical students, most of whom were Christian. Similarly, we found that the attitudes of participants toward abortion were the strongest predictors of their willingness to provide safe abortion services.

Considering religion, the Muslim participants were less likely to agree to provide safe abortion services than the Buddhist participants. Religion might affect the decision-making process related to provision of safe abortion services [17, 28, 37, 38]. According to the Islamic doctrine, Muslims doing something against their principles will be penalized; this might be the reason that Muslims were less willing than Buddhists to provide safe abortion services. Half the number of participants unwilling to perform an abortion were prepared to refer pregnant women for safe abortion services, while the other half considered advising the women to continue their pregnancies. The denial of the provision of safe abortion services may have serious consequences on women’s health and well-being and is a risk factor for seeking unsafe, illegal abortion methods that can have serious health consequences [39]. However, we found that only 41.3% of the participants willing to refer pregnant women for safe abortion services knew where to send them. In Thailand, there is no organized system to aid healthcare providers in referring pregnant women for safe abortion services. Therefore, improving and publicizing the referral system should be considered to reduce the rates of unsafe abortion procedures.

The results section shows that nurses agreed to provide safe abortion services for certain conditions, such as the maternal health or fetal anomalies. In contrast, most nurses disagree with providing services for a socioeconomic condition, the most common condition that causes pregnant women to seek abortions [40]. Another issue is that under the conditions that nurses in this study disagree with providing safe abortion services, most did not know where they should refer women for abortion.

Through this survey, we received a robust response that represents the attitude of registered nurses in tertiary hospitals of the area. The evaluation of responses from practicing nurses can provide detailed information regarding the current healthcare system. Furthermore, our questionnaires were tested for validity and reliability and were electronically distributed to ensure the anonymity of the participants.

Nevertheless, this study had a few limitations. First, the data were collected from a single hospital in the southernmost province of Thailand. However, the study setting has the largest Muslim population in Thailand, and this center is the largest referral center in the area. The results cannot be generalized to those of other regions in Thailand, particularly when considering differences in religious practices between these regions. However, the results of this study may also be applied to the nearby South East Asian countries, which share similar characteristics in terms of Muslims living in Buddhist-dominated countries and having similar Asian cultures [41]. Hence, further research involving participants from varied regions and practicing different religious beliefs is required.

Second, this was a survey based on self-administered questionnaires, and the information provided was not elaborate. Considering abortion is an extremely sensitive issue, in-depth interviews may provide researchers with more details to understand the reasons behind the attitude of participants toward abortion. Third, some departments had only a few nurses, and to maintain anonymity, a clear mention of only the number of nurses working in the OB-GYN department was made; the rest were included as part of other unspecified departments. Further studies at multiple sites including nurses practicing in relevant departments, such as pediatrics and psychiatry, should be carried out.

## Conclusions

Nurses in the southernmost province of Thailand lack knowledge of the amended abortion act and do not have a favorable attitude toward abortion. Compared with Buddhist participants, Muslim participants have a less favorable attitude toward abortion and the intentions behind it as well as less willingness to provide abortion services. Participants with higher knowledge scores had a more favorable moral attitude toward abortion than those with lower knowledge scores, and those with a favorable attitude toward abortion were more likely to provide abortion services. Enhancing the knowledge regarding abortion laws and practices among nurses may improve their attitude toward abortion and positively influence the healthcare system for women.

## Supplementary Information

**Additional file 1: Supplemental Table 1.** Moral attitude of participants toward abortion by religion. **Supplemental Table 2.** Moral attitude of participants toward intended practice of abortion service by religion. **Supplemental Table 3.** Willingness to provide abortion services (to help doctor perform abortion) by religion.

## Data Availability

The datasets used and analyzed during the current study available from the corresponding author on reasonable request.

## Abbreviations

SD: standard deviation
OB-GYN: obstetrician-gynecologist
