# Supplementary material for: Survey of knowledge and attitude regarding induced abortion among nurses in a tertiary hospital in Thailand after amendment of the abortion act: a cross-sectional study
Source: BMC Womens Health. 2022 Nov 18;22:454. doi: 10.1186/s12905-022-02064-7 (PMC9673310; doi:10.1186/s12905-022-02064-7)
Supplement: Supplementary file 1 — Additional file 1: Supplemental Table 1. Moral attitude of participants toward abortion by religion. Supplemental Table 2. Moral attitude of participants toward intended practice of abortion service by religion. Supplemental Table 3. Willingness to provide abortion services (to help doctor perform abortion) by religion. [file 12905_2022_2064_MOESM1_ESM.docx]

**Supplemental Table 1** Moral attitude of participants toward abortion by religion (N = 375)

|  | **Strongly Agree** | | **Agree** | | **Neutral** | | **Disagree** | | **Strongly Disagree** | |
| --- | --- | --- | --- | --- | --- | --- | --- | --- | --- | --- |
|  | **Muslim**  **n (%)** | **Buddhist**  **n (%)** | **Muslim**  **n (%)** | **Buddhist**  **n (%)** | **Muslim**  **n (%)** | **Buddhist**  **n (%)** | **Muslim**  **n (%)** | **Buddhist**  **n (%)** | **Muslim**  **n (%)** | **Buddhist**  **n (%)** |
| **Pro-choice** |  |  |  |  |  |  |  |  |  |  |
| 1. Abortion can be a good thing in any circumstances. | 6  (2.7) | 6  (3.9) | 5  (2.3) | 8  (5.2) | 17  (7.7) | 14  (9.1) | 61 (27.6) | 53  (34.4) | 132 (59.7) | 73 (47.4) |
| 2. Abortion is a woman’s right. | 15  (6.8) | 22  (14.3) | 46 (20.8) | 50  (32.5) | 21  (9.5) | 19  (12.3) | 63 (28.5) | 32  (20.8) | 76 (34.4) | 31 (20.1) |
| 3. Abortion after gestational age 12 week is acceptable in every situation. | 8  (3.6) | 8  (5.2) | 19  (8.6) | 17  (11.0) | 37 (16.7) | 29  (18.8) | 71 (32.1) | 45  (29.2) | 86 (38.9) | 55 (35.7) |
| **Pro-life** |  |  |  |  |  |  |  |  |  |  |
| 4. Abortion is the same as murder. | 87 (39.4) | 33  (21.4) | 55 (24.9) | 40  (26.0) | 44 (19.9) | 40  (26.0) | 29 (13.1) | 24  (15.6) | 6  (2.7) | 17 (11.0) |
| 5. Abortion is wrong. | 88 (39.8) | 37  (24.0) | 55  (24.9) | 47  (30.5) | 40 (18.1) | 27  (17.5) | 26 (11.8) | 31  (20.1) | 12  (5.4) | 12 (7.8) |
| 6. Abortion is sinful | 120 (54.3) | 49  (31.8) | 54 (24.4) | 51  (33.1) | 24 (10.9) | 26  (16.9) | 18  (8.1) | 17  (11.0) | 5  (2.3) | 11 (7.1) |
| **Conditional agreement** |  |  |  |  |  |  |  |  |  |  |
| 7. Abortion after gestational age 12 week is acceptable in some situation. | 39 (17.6) | 33  (21.4) | 88 (39.8) | 69  (44.8) | 51 (23.1) | 26  (16.9) | 25 (11.3) | 14  (9.1) | 18  (8.1) | 12 (7.8) |

**Supplemental Table 2** Moral attitude of participants toward intended practice of abortion service by religion (N = 375)

| **Gestational age under 12 weeks** | **Strongly Agree** | | **Agree** | | **Neutral** | | **Disagree** | | **Strongly Disagree** | |
| --- | --- | --- | --- | --- | --- | --- | --- | --- | --- | --- |
|  | **Muslim**  **n (%)** | **Buddhist**  **n (%)** | **Muslim**  **n (%)** | **Buddhist**  **n (%)** | **Muslim**  **n (%)** | **Buddhist**  **n (%)** | **Muslim**  **n (%)** | **Buddhist**  **n (%)** | **Muslim**  **n (%)** | **Buddhist**  **n (%)** |
| **Physical health** |  |  |  |  |  |  |  |  |  |  |
| 1.The pregnant woman has a serious physical disease(s). | 94 (42.5) | 57  (37.0) | 76 (34.4) | 66  (42.9) | 29  (13.1) | 19  (12.3) | 15  (6.8) | 7  (4.5) | 7  (3.2) | 5  (3.2) |
| 2. The pregnant woman has HIV/ AIDS. | 35 (15.8) | 36  (23.4) | 32 (14.5) | 34  (22.1) | 61  (27.6) | 27  (17.5) | 51  (23.1) | 35  (22.7) | 42  (19.0) | 22  (14.3) |
| **Mental Health** |  |  |  |  |  |  |  |  |  |  |
| 3. The pregnant woman has a serious mental disease(s). | 84 (38.0) | 56  (36.4) | 76 (34.4) | 61  (39.6) | 34  (15.4) | 19  (12.3) | 17  (7.7) | 11  (7.1) | 10  (4.5) | 7  (4.5) |
| 4. The fetus has a serious defect that makes it nonviable. | 117 (52.9) | 83  (53.9) | 61 (27.6) | 51  (33.1) | 21  (9.5) | 9  (5.8) | 14  (6.3) | 5  (3.2) | 8  (3.6) | 6  (3.9) |
| 5. The fetus has a serious defect but will be viable and being handicapped. | 81 (36.7) | 74  (48.1) | 60 (27.1) | 55  (35.7) | 47  (21.3) | 12  (7.8) | 19  (8.6) | 7  (4.5) | 14  (6.3) | 6  (3.9) |
| **Sexual assault** |  |  |  |  |  |  |  |  |  |  |
| 6. The woman has become pregnant from rape | 68 (30.8) | 66  (42.9) | 56 (25.3) | 46  (29.9) | 45  (20.4) | 24  (15.6) | 30  (13.6) | 11  (7.1) | 22  (10.0) | 7  (4.5) |
| 7. The woman has become pregnant through intercourse between blood relatives. | 35 (15.8) | 37  (24.0) | 37 (16.7) | 35  (22.7) | 58  (26.2) | 48  (31.2) | 46  (20.8) | 21  (13.6) | 45  (20.4) | 13  (8.4) |
| **Socioeconomic Problems** |  |  |  |  |  |  |  |  |  |  |
| 8. The pregnant woman is under age 20. | 3  (1.4) | 11  (7.1) | 12  (5.4) | 19  (12.3) | 35  (15.8) | 29  (18.8) | 79  (35.7) | 51  (33.1) | 92  (41.6) | 44  (28.6) |
| 9. The pregnant woman is under age 15. | 18  (8.1) | 17  (11.0) | 20  (9.0) | 26  (16.9) | 39  (17.6) | 35  (22.7) | 71  (32.1) | 39  (25.3) | 73  (33.0) | 37  (24.0) |
| 10. The pregnant women that the man refused to be the father of the child. | 1  (0.5) | 6  (3.9) | 13  (5.9) | 18  (11.7) | 28  (12.7) | 32  (20.8) | 78  (35.3) | 47  (30.5) | 101  (45.7) | 51  (33.1) |
| 11. The pregnant women that the man refused to marry the pregnant women. | 2  (0.9) | 4  (2.6) | 10  (4.5) | 15  (9.7) | 29  (13.1) | 36  (23.4) | 79  (35.7) | 48  (31.2) | 101  (45.7) | 51  (33.1) |
| 12.The couples that already have enough children. | 2  (0.9) | 6  (3.9) | 9  (4.1) | 17  (11.0) | 25  (11.3) | 29  (18.8) | 77  (34.8) | 45  (29.2) | 108  (48.9) | 57  (37.0) |
| 13.The woman has become pregnant from contraceptive failure. | 5  (2.3) | 14  (9.1) | 11  (5.0) | 19  (12.3) | 27  (12.2) | 30  (19.5) | 84  (38.0) | 43  (27.9) | 94  (42.5) | 48  (31.2) |

| **Gestational age over 12 weeks** | **Strongly Agree** | | **Agree** | | **Neutral** | | **Disagree** | | **Strongly Disagree** | |
| --- | --- | --- | --- | --- | --- | --- | --- | --- | --- | --- |
|  | **Muslim**  **n (%)** | **Buddhist**  **n (%)** | **Muslim**  **n (%)** | **Buddhist**  **n (%)** | **Muslim**  **n (%)** | **Buddhist**  **n (%)** | **Muslim**  **n (%)** | **Buddhist**  **n (%)** | **Muslim**  **n (%)** | **Buddhist**  **n (%)** |
| **Physical health** |  |  |  |  |  |  |  |  |  |  |
| 1.The pregnant woman has a serious physical disease(s). | 90 (40.7) | 66  (42.9) | 65 (29.4) | 54  (35.1) | 30  (13.6) | 18  (11.7) | 18  (8.1) | 9  (5.8) | 18  (8.1) | 7  (4.5) |
| 2. The pregnant woman has HIV/ AIDS. | 45 (20.4) | 39  (25.3) | 39 (17.6) | 39  (25.3) | 30  (13.6) | 23  (14.9) | 62  (28.1) | 31  (20.1) | 45  (20.4) | 22  (14.3) |
| **Mental Health** |  |  |  |  |  |  |  |  |  |  |
| 3. The pregnant woman has a serious mental disease(s). | 78 (35.3) | 58  (37.7) | 63 (28.5) | 60  (39.0) | 31  (14.0) | 19  (12.3) | 28  (12.7) | 8  (5.2) | 21  (9.5) | 9  (5.8) |
| 4. The fetus has a serious defect that makes it nonviable. | 100 (45.2) | 79  (51.3) | 66 (29.9) | 57  (37.0) | 25  (11.3) | 7  (4.5) | 17  (7.7) | 4  (2.6) | 13  (5.9) | 7  (4.5) |
| 5. The fetus has a serious defect but will be viable and being handicapped. | 80 (36.2) | 71  (46.1) | 54 (24.4) | 57  (37.0) | 45  (20.4) | 13  (8.4) | 22  (10.0) | 6  (3.9) | 20  (9.0) | 7  (4.5) |
| **Sexual assault** |  |  |  |  |  |  |  |  |  |  |
| 6. The woman has become pregnant from rape | 60 (27.1) | 55  (35.7) | 42 (19.0) | 49  (31.8) | 50  (22.6) | 24  (15.6) | 39  (17.6) | 13  (8.4) | 30  (13.6) | 13  (8.4) |
| 7. The woman has become pregnant through intercourse between blood relatives. | 23 (10.4) | 32  (20.8) | 42 (19.0) | 27  (17.5) | 48  (21.7) | 48  (31.2) | 54  (24.4) | 27  (17.5) | 54  (24.4) | 20  (13.0) |
| **Socioeconomic Problems** |  |  |  |  |  |  |  |  |  |  |
| 8. The pregnant woman is under age 20. | 1  (0.5) | 12  (7.8) | 11  (5.0) | 14  (9.1) | 23  (10.4) | 26  (16.9) | 90  (40.7) | 57  (37.0) | 96  (43.4) | 45  (19.2) |
| 9. The pregnant woman is under age 15. | 12  (5.4) | 14  (9.1) | 20  (9.0) | 21  (13.6) | 28  (12.7) | 42  (27.3) | 79  (35.7) | 41  (26.6) | 82  (37.1) | 36  (23.4) |
| 10. The pregnant women that the man refused to be the father of the child. | 2  (0.9) | 6  (3.9) | 9  (4.1) | 20  (13.0) | 24  (10.9) | 27  (17.5) | 91  (41.2) | 49  (31.8) | 95  (43.0) | 52  (33.8) |
| 11. The pregnant women that the man refused to marry the pregnant women. | 2  (0.9) | 6  (3.9) | 10  (4.5) | 19  (12.3) | 24  (10.9) | 29  (18.8) | 92  (41.6) | 49  (31.8) | 93  (42.1) | 51  (33.1) |
| 12.The couples that already have enough children. | 3  (1.4) | 9  (5.8) | 9  (4.1) | 16  (10.4) | 20  (9.0) | 22  (14.3) | 94  (42.5) | 51  (33.1) | 95  (43.0) | 56  (36.4) |
| 13.The woman has become pregnant from contraceptive failure. | 4  (1.8) | 12  (7.8) | 9  (4.1) | 24  (15.6) | 29  (13.1) | 27  (17.5) | 87  (39.4) | 44  (28.6) | 92  (41.6) | 47  (30.5) |

**Supplemental Table 3** Willingness to provide abortion services (to help doctor perform abortion) by religion (N = 375)

|  | **Muslim**  **n (%)** | **Buddhists**  **n (%)** | **Total**  **N (%)** |
| --- | --- | --- | --- |
| **Willingness to perform abortion in unintended pregnancy**  Yes  For both medical and surgical abortion  Only for medical abortion  Only for surgical abortion  No | 40 (18.1)  21 (9.5)  16 (7.2)  3 (1.4)  181 (81.9) | 40 (26.0)  22 (14.3)  13 (8.4)  5 (3.2)  114 (74.0) | 80 (21.3)  43 (11.5)  29 (7.7)  8 (2.1)  295 (78.7) |
| **Management approach in those who were not willing to provide abortion service**  Advice women to continue pregnancy and seek adoption  Advice women to continue pregnancy and raise their own child  Refer pregnant women for safe abortion | 87 (48.1)  26 (14.4)  68 (37.6) | 39 (34.2)  8 (7.0)  67 (58.8) | 126 (33.6)  34 (9.1)  135 (36.0) |
| **Knowing where to refer pregnant women for safe abortion**  Yes  No | 86 (38.9)  135 (61.1) | 69 (44.8)  85 (55.2) | 155 (41.3)  220 (58.7) |
